# Micro-Computed Tomographic Analysis of the Shaping Ability of XP-Endo Shaper in Oval-Shaped Distal Root Canals of Mandibular Molars

**DOI:** 10.14744/eej.2021.44153

**Published:** 2021-11-19

**Authors:** Ane POLY, Wei-Ju Louis TSENG, Fernando MARQUES, Frank Carsten SETZER, Bekir KARABUCAK

**Affiliations:** 1. Department of Integrated Clinical Procedures, School of Dentistry, Rio de Janeiro State University, Rio de Janeiro, Brazil; 2. Department of Orthopaedic Surgery, McKay Orthopaedic Research Laboratory, Perelman School of Medicine, University of Pennsylvania, Philadelphia, PA, USA;Department of Medicine, Center for Translational Medicine, Sidney Kimmel Medical College, Thomas Jefferson University, Philadelphia, PA, USA; 3. Department of Endodontics, School of Dental Medicine, University of Pennsylvania, Philadelphia, USA

**Keywords:** Dental instruments, endodontics, root canal preparation, X-Ray microtomography

## Abstract

**Objective:**

To compare the shaping ability of the XP-endo Shaper (XPS) system to the ProTaper Next (PTN) system in oval-shaped distal root canals.

**Methods:**

From 12 mandibular molars, distal roots with moderately curved single oval canals were randomly assorted to be instrumented with XPS (experimental group) or PTN (control group) and then scanned using micro-computed tomography [Scan 1]. The root canals of the XPS samples were prepared following the manufacturer's instructions using 15 insertions (XPS15) and rescanned [Scan 2]. An additional 10 insertions to the working length were applied, totalling 25 insertions (XPS25), and the roots were rescanned again [Scan 3]. PTN samples were prepared up to the X3 instrument (PTNX3) and rescanned [Scan 2]. The dentine removed and the unprepared areas were assessed. Data were analysed using a t-test with significance at α=0.05.

**Results:**

XPS25 was associated with a significantly greater dentine removal than XPS15 over the entire root canal length and in all three-thirds of the root canal (P<0.05). XPS25 significantly removed more dentine than PTNX3 in only the coronal third (P<0.05). XPS25 was also associated with a significantly smaller percentage of unprepared areas than XPS15 overall and in the coronal third (P<0.05). PTNX3 was associated with a significantly larger percentage of unprepared areas than XPS15 and XPS25 overall and in the coronal and middle thirds (P<0.05).

**Conclusion:**

Ten additional movements with XPS significantly improved instrumentation capacity, reducing the percentage of untouched surface areas but also removing more dentine.

## Introduction

Evidence based dentistry does not support making that strict causation argument for bacterial reduction in the root canal system and elimination of apical periodontitis (1). Microbes may colonise root canal systems as planktonic microorganisms by invading dentinal tubules (2-4) or attached to the walls as a biofilm. Biofilm is an important component in forming apical periodontitis and persistent post-treatment disease (5, 6). Mechanical instrumentation serves two purposes when treating infected root canal systems. First, mechanical instrumentation enables widening of the canal dimensions to allow penetration of irrigation solutions, such as sodium hypochlorite. Second, mechanical instrumentation directly disinfects by removing layers of infected dentine and removing biofilms from root canal wall surfaces, which may be resistant to chemical means of disinfection (7).

**Highlights**

• This study provided data on the shaping ability of XP-endo Shaper following the manufacturer's instructions of use (XPS15) compared to an additional 10 long movements with the instrument (XPS25).
• XPS25 removed significantly more amount of dentine than XPS15 overall and in the three thirds.
• When compared to XPS15, XPS25 led to a significantly smaller percentage of unprepared areas overall and in the coronal.

A large percentage of root canals are oval in cross-section (8, 9). However, endodontic files predominantly used today shape canals round and do not follow the original cross-sectional anatomy (10). The shaping of oval canals may lead to either under-preparation of the wide oval dimension, leaving behind unprepared areas (10 PAQUE et al). Consequently, over-preparation of the narrow oval dimension may result in sacrificing unnecessary tooth structure. Despite the potentially excessive dentine removal, Paqué et al. (11) showed that a 25/.06 preparation with round files in oval canals resulted in as much as 80% of the canal walls remaining untouched.

To overcome the limitations of traditional rotary file systems, the XP-endo Shaper (XPS; FKG, La Chaux-de-Fonds, Switzerland) was introduced, able to contact more of the canal wall surface (12). This instrument is heat-treated and features a serpentine-shaped design with a tip size of 27 and a taper .01, as well as a semi-active cutting tip. This design provides high flexibility and the ability to contract and expand according to the natural cross-sectional shape of the canal (13). The instrument is in the Martensite phase at room temperature (~20°C) but changes its form to the pronounced serpentine shape at ~35°C, as in the oral environment (14). At body temperature, it also shifts to a predominantly Austenite phase (14). Depending on the resistance to expansion in the canal and the time it is used during root canal preparation, the file can extend the apical preparation size beyond its actual tip size and the taper of the instrumentation adaptive according to the original canal anatomy (15). According to the manufacturer, depending on the original anatomy, apical canal sizes of up to 90 and natural tapers of up to 0.08 can be reached by XPS without resistance. According to the manufacturer, the full XPS protocol requires a two step instrumentation with XPS after glide path preparation and working length determination (13). The first step of instrumentation with XPS progresses to working length (13). The second step requires the application of additional strokes with the same XPS instrument for canal enlargement and adaptive increase of the preparation taper, determined by the original canal diameter and shape (13).

Azim et al. (16) demonstrated that the XPS instrument was more effective in preparing canal surfaces than conventional round nickel-titanium (NiTi) rotary systems. Moreover, the final taper of the preparation was influenced by the original anatomy of the treated tooth and less by the instrument's geometry (16). According to the manufacturer, its design allows the file to reach working length (WL) using long amplitude vertical movements after glide path establishment. Once the WL is reached, 15 long-stroke movements should be applied for adequate canal shaping (13).

Technology that offers better shaping abilities while maintaining the root canal anatomy is potentially advantageous for preventing and treating apical periodontitis. Revised protocols have been introduced by the manufacturer intending to enhance the shaping efficiency of XPS (13). When operated at a higher speed (3000 rpm), XPS has shown to be more efficient in gutta-percha removal (17). Likewise, high-speed preparation without a glide path has been shown to be a more efficient protocol for shaping curved canals with XPS (18). Another protocol suggested that extending the activation time of XPS at the WL increases its shaping efficiency in round canals (19).

However, no information is available in the literature on whether supplementary, vertical movements with the XPS instrument, in addition to the manufacturer's protocol, enhance its shaping ability in oval-shaped root canals.

Therefore, this study aimed to investigate the ability of the XPS to instrument oval distal canals of mandibular molars with supplementary 10 long vertical movements and compare its performance with the manufacturer's recommended protocol using micro-computed tomographic (micro-CT) analysis. The ProTaper Next system (PTN; Dentsply Sirona, Tulsa, OK, USA) was used as the reference for comparison. The null hypotheses tested were that:

1. There would be no differences between the suggested protocol and the manufacturer's recommended protocol regarding the dentine removed. And,
2. There would be no differences between the suggested protocol and the manufacturer's recommended protocol regarding the unprepared area.

## Materials and Methods

### Sample selection

The effect size was estimated at 2.4 based on the results of a previous study (16). Assuming an alpha-type error of.05 and a power beta of.95, a total of 12 specimens were determined as the sample size to run the paired t-test to observe significant differences (G*Power 3.1 software; Heinrich-Heine-University, Düsseldorf, Germany).

After local Ethics Committee approval [#62903316.3.0000.5259; 5^th^ May 2017], permanent mandibular molars with distal roots with fully formed apex were included. In addition, previously treated canals and roots with dentinal defects at the external aspect were excluded. After this pre-selection, a pool of extracted human mandibular molars was inspected under high magnification (24x; ZEISS ProErgo, Carl Zeiss Meditec, Dublin, CA, USA), and only teeth free of fractures, cracks, other cemental-dentinal defects, or artificial alterations were chosen.

The mesial roots were sectioned to prevent confounding variables, and the teeth were decoronated 3 mm above the cementoenamel junction (CEJ). Periapical radiographs of the distal roots were taken in the buccolingual and mesiodistal directions (Carestream CS2200, Atlanta, GA, USA) to detect single canals (Vertucci type I configuration) and any possible root canal obstruction. The radiographs were imported to AutoCAD 2015 (Autodesk, San Rafael, CA, USA), and the curvatures were measured. The distal roots of moderate curvature (10°-20°) (20) were included. The oval canal anatomy was defined by a ratio greater than 2, located 6 mm from the apex (21), and later verified using the structure model index (SMI; see below). Apical patency of the canals was confirmed by a size 10 K-file (Dentsply Sirona).

Specimens were scanned using a micro-CT system (vivaCT-40; Scanco Medical, Bassersdorf, Switzerland) at 21 μm nominal voxel size. The scanner was used at 55 kVp energy and 145 μA intensity with an integration time of 200 ms. The acquired projection images were reconstructed, and the preoperative three-dimensional (3D) models of the distal root canals were rendered by the Scanco software (Scanco Medical). A collection of 133 distal roots of mandibular molars with single oval canals and a moderate curvature were chosen.

**Figure 1.**
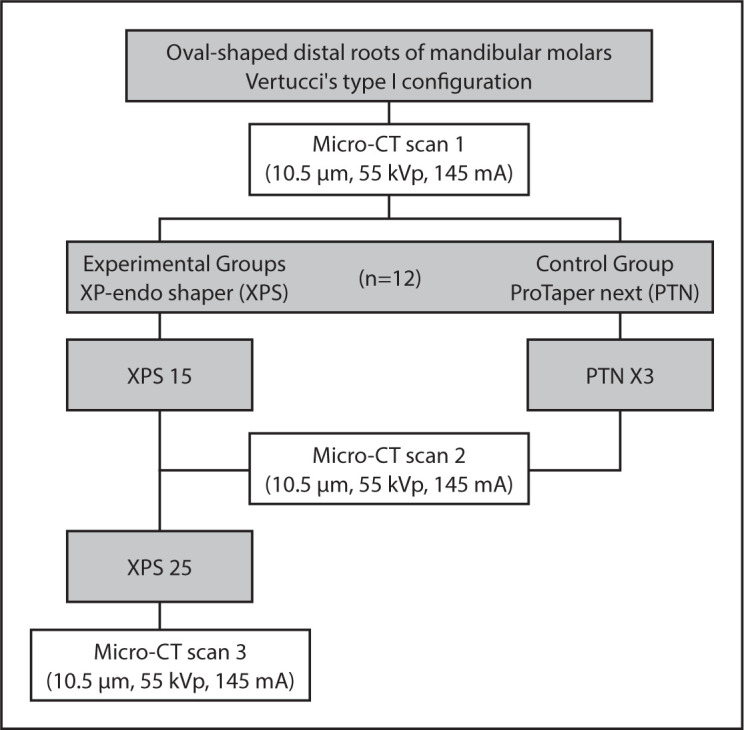
Flowchart of the experimental procedures

The initial values for volume and surface area were calculated using the Scanco software. The volume and surface area of interest were selected, extending from the furcation level to the apex of the root. Next, 12 samples with similar anatomical structures were selected, and the normality of the morphological parameters (length, curvature, apical size at WL, SMI, roundness, surface area, volume) was verified. Specimens were then randomly assigned, using a randomisation algorithm from www.random.org to XPS or PTN groups (Fig. 1).

### Root canal preparation

The WL was determined by reducing 0.5 mm from the overall canal length. A glide path was established using a stainless-steel size 15 K-file (Dentsply Sirona) to WL. Apical foramina were sealed with Opaldam (Ultradent, South Jordan, UT, USA) to create a closed-end system. During canal instrumentation, each tooth was immersed in a warm water bath (37^°^C) up to the CEJ to mimic clinical conditions at intra-oral temperature. Samples were divided into 2 groups and prepared according to the following protocols:

### XPS (experimental groups)

XPS15: Following the manufacturer's instructions, the XPS file was inserted into the root canal. A total of up to 5 movements (5-7 mm amplitude) were applied until the file reached the WL. Once the file reached WL, the canals were further instrumented with 15 long movements to WL (7-9 mm amplitude). This was followed by the second acquisition of micro-CT scans in the XPS group.

XPS25: Canals were further instrumented with additional 10 long movements to WL (for a total of 25 movements after WL had been reached). This was followed by the third acquisition of micro-CT scans in the XPS group.

### PTNX3 (control group)

ProTaper Next is a variable taper file system, with the taper progressively increasing towards the file tip, and an off-centered, rectangular cross section. Following the manufacturer's instructions, root canals were instrumented in a pecking motion to WL by using the PTN system as follows: PTN X1 (yellow; 17/.04v) followed by X2 (red; 25/.06v) and X3 (blue; 30/.07v). This was followed by the second acquisition of micro-CT scans in the PTN group.

A single, experienced operator prepared all root canals using a ProMark Endo Motor (Dentsply Sirona). XPS files were operated at 900 rpm with 1 Ncm torque. PTN instruments were operated at 300 rpm and 520 gcm. During instrumentation, each canal was irrigated with 2 mL of 5% sodium hypochlorite using 30 ga NaviTip® irrigation needles (Ultradent) into the apical 3 mm between each filing interval. With the complete instrumentation, each canal was rinsed with an additional 1 mL of 5% NaOCl followed by 1 mL of 17% EDTA using 30 ga NaviTip® irrigation needles at the apical 1 mm.

Preparation time for each canal was recorded in seconds using a digital chronometer, including mechanical instrumentation and excluding irrigation time. Before each micro-CT scanning, canals were dried with absorbent paper points, and scanning was performed applying the same parameter settings described above.

### Image processing and analysis

^Postoperative 3D models of the samples were reconstructed by the micro-CT system and then co-registered with the preoperative scan, using the rigid registration module of 3DSlicer 4.6.2 (3DSlicer, http://www.slicer.org) (22). Registered images of each root were imported into Fiji v.1.46r (ImageJ, Madison, WI, USA) (23) to determine the dentine volume removed after root canal preparation by subtracting pre- and postoperative dentine using morphologic operations.^

The percentage of the unprepared area was calculated in relation to the original canal area (total number of surface voxels) by dividing the number of static surface voxels by the total number of surface voxels (24, 25). Data from the root canal images extending from the CEJ to the apex were calculated for the entire canal (overall) and by thirds (coronal, middle, and apical) as used elsewhere (16, 26).

### Statistical analysis

The assumptions of normality for the morphological parameters were confirmed by the Shapiro-Wilk test (P>0.05). Changes in the parameters and the amount of dentine removed, percentage of unprepared area, and time spent during instrumentation among the experimental groups (XPS15, XPS25), were compared using the paired t-test. The same parameters were evaluated between the experimental and control groups (PTNX3) using the unpaired t-tests (Prism 6.2; GraphPad Software Inc., San Diego, USA). The significance level for all analyses was 5%.

## Results

There were no significant differences before preparation for all variables between groups after the random distribution (P>0.05), indicating homogeneity in the data set (Table 1). Table 2 describes the postoperative data of SMI, roundness, surface area, volume, dentine removed, and unprepared area for the overall canal length and each third of the root canals.

**Table 1.** Preoperative morphometric data (mean±standard deviation) of the samples from XP-endo Shaper and ProTaper Next groups

	XPS	PTN
Length (mm)	14.50±1.41	15.58±0.66
Curvature (o)	16.33±3.01	15.33±2.50
Canal taper at the WL (mm)	0.17±0.07	0.19±0.03
SMI	2.16±0.39	2.13±0.41
Roundness (mm)	0.43±0.15	0.38±0.17
Surface area (mm^2^)	65.78±22.54	84.93±38.6
Volume (mm^3^)	5.91±3.09	9.57±7.39

XPS, XP-endo Shaper; PTN, ProTaper Next; WL, Working Length. SMI values vary from 1 indicating more parallel plates to 4 a perfect ball. There were no statistical differences between groups (P>0.05; unpaired t-test)

**Table 2.** Morphometric data (Mean±standard deviation) for distal oval root canals in mandibular molars prepared with XP-endo Shaper and ProTaper Next systems

	XPS15	XPS25	PTNX3
Overall			
Δ SMI	0.14±0.04^A^	0.26±0.11^A^	0.17±0.10
Δ Roundness (mm)	0.14±0.09A*	0.16±0.10^B^^*^	0.06±0.06^*^
Δ Surface area (%)	11.23±5.82^A^	17.52±9.44^B^	6.67±9.03
Δ Volume (%)	48.63±27.22^A^	58.38±30.66^B^	29.43±19.62
Dentine removed (mm^3^)	2.95±1.83^A^	3.54±2.02^B^	1.56±0.63
Unprepared area (%)	4.13±3.38^A^^*^	2.35±2.03^B^^*^	16.85±7.75^*^
Coronal third			
Δ SMI	0.21±0.11^A^^*^	0.29±0.08^B^^*^	0.06±0.03^*^
Δ Roundness (mm)	0.10±0.05^A^	0.12±0.05^B^	0.05±0.04
Δ Surface area (%)	6.46±3.54^A^	10.69±4.13^B^^*^	3.52±2.65^*^
Δ Volume (%)	34.40±16.06^A^	43.92±19.35^B^^*^	17.95±11.59^*^
Dentine removed (mm^3^)	1.29±0.77^A^	1.64±0.88^B^^*^	0.65±0.35*
Unprepared area (%)	3.12±2.44^A^^*^	1.19±0.99^B^^*^	13.66±6.13^*^
Middle third			
Δ SMI	0.15±0.09^A^	0.24±0.09^A^	0.25±0.16
Δ Roundness (mm)	0.15±0.09^A^	0.18±0.11^A^	0.09±0.09
Δ Surface area (%)	8.73±5.85^A^	16.18±9.79^B^	9.52±11.50
Δ Volume (%)	68.77±49.82^A^	79.99±55.88^B^	52.40±42.93
Dentine removed (mm^3^)	1.15±0.74^A^	1.34±0.81^B^	0.66±0.26
Unprepared area (%)	3.68±3.03^A^^*^	1.95±1.39^A^^*^	19.21±10.55^*^
Apical third			
Δ SMI	0.15±0.11^A^	0.31±0.24^A^	0.11±0.15
Δ Roundness (mm)	0.18±0.12^A^	0.19±0.13^A^^*^	0.05±0.05^*^
Δ Surface area (%)	35.76±27.93^A^	47.47±37.11^B^	22.63±41.14
Δ Volume (%)	95.76±89.22^A^	103.70±91.26^A^	42.94±27.28
Dentine removed (mm^3^)	0.51±0.33^A^	0.57±0.33^B^	0.25±0.08
Unprepared area (%)	5.74±5.56^A^	5.10±4.89^A^	22.52±20.54

Δ, variation; XPS, XP-endo Shaper; PTN, ProTaper Next. Experimental groups: XPS15 and XPS25. Control group: PTNX3. Different letters represent a statistically significant difference among experimental groups (P<0.05, paired t-test). *Indicates a statistically significant difference between experimental and control groups (P<0.05, unpaired t-test)

Significant differences in the amount of dentine removed were seen between XPS15 and XPS25 overall and in the three thirds (P<0.05). In addition, between the experimental groups and the control group, there was significant difference between XPS25 and PTNX3 in the coronal third (P<0.05).

**Figure 2.**
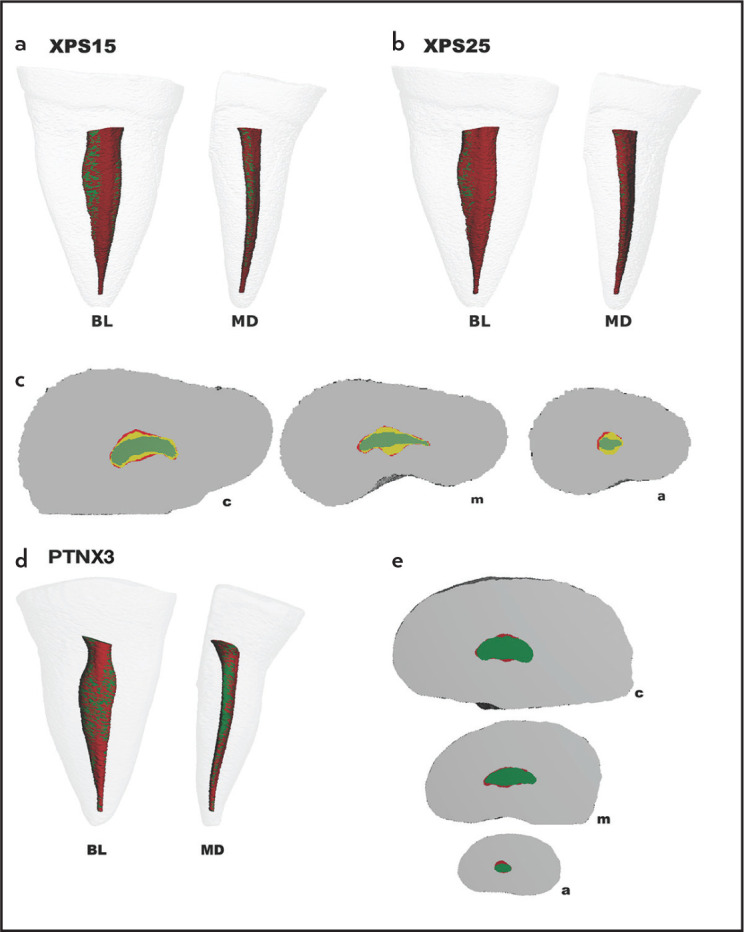
Representative 3D reconstructions of the internal anatomy of samples. Buccolingual (BL) and mesiodistal (MD) views of superimposed specimens before (green) and after preparation (red): XPS15 (a), XPS25 (b), and PTNX3 (d). Cross-section views of the superimposed root canals before (green) and after preparations: XPS15 (yellow) and XPS25 (red) (c), and PTNX3 (red) (E), at the coronal (c), middle (m) and apical (a) thirds

There was a significant difference in the percentage of unprepared areas between XPS15 and XPS25 overall and the coronal third (P<0.05). Significant differences were seen only in the apical third between the experimental and control groups (P>0.05). Figure 2 shows representative 3D reconstructions of the internal anatomy of XPS and PTNX3 samples before and after preparation.

Statistically significant differences were observed for the time spent preparing the root canals between XPS15 (90±7 s) and XPS25 (116±8 s) and between XPS15 and PTNX3 (112±9 s) (P<0.05).

## Discussion

The present study evaluated the shaping ability of the off-centred rotation axis single-file XPS system applying 10 extra movements during instrumentation of oval-shaped distal canals from mandibular molars. Furthermore, the preparation with the XPS was compared to the off-centred rotation axis multi-file PTN system as a control group using micro-CT technology.

The choice of the PTN system as the control group was based on the fact that PTN is a well-known system used by many clinicians. Moreover, PTN instruments have an offset mass of rotation that creates an asymmetrical rotary motion so that at any given cross-section, the file only contacts the wall at two points (27). This asymmetry allows the PTN instrument to cut a bigger envelope of motion than a similarly sized file with symmetrical mass and rotation axis (28). According to the XPS manufacturer, the instrumentation protocol would lead to at least a 30/.04 preparation, but allow to expand up to .08 taper in a natural canal anatomy. Thus, the PTN X3 instrument (30/.07v) was chosen for comparison.

Depending on the original canal morphology, preparation to larger tapers may lead to greater dentine removal. In general, remaining dentine positively influences a tooth's resistance to fracture (29). In the present study, the dentine removal was significantly higher in XPS25 than XPS15 and, XPS25 significantly removed more dentine than PTNX3 in the coronal third. Therefore, the first null hypothesis was rejected since XPS25 prepared significantly more dentine than XPS15.

De-Deus et al. (19) evaluated the action of XPS at WL, applying the instrument for 15, 30, and 45 seconds longer than the manufacturer had recommended. They concluded that an extra 45s using XPS at the WL resulted in a better shaping efficiency of the instrument. However, instead of comparing time, for the present study, it was decided to evaluate the number of XPS movements. The number of movements was based on the manufacturer's instructions that also advise on the number of movements, rather than time. Therefore, using the number of movements allows for a better comparison of the protocols and a more efficient and reliable manner of conducting the experiments. Nevertheless, the time of instrumentation was also recorded to allow comparisons with other studies. Moreover, the manufacturer's instructions clearly state that the instrument should be kept spinning and moving in the canal, which adds up to a total operation time (13).

The quest for improving chemical and mechanical disinfection performances is ongoing. Micro-CT analysis has revealed that unprepared areas of root canals contain remnants of pulp tissue and bacteria, especially in the apical third (7). In the present study, the XPS15 had a greater percentage of unprepared areas than XPS25 overall and in the coronal third, leading to rejection of the second null hypothesis. Further, both experimental groups showed a smaller percentage of unprepared areas than PTNX3, except in the apical third, highlighting the increased efficacy of XPS in the coronal and middle canal sections that are known to exhibit more oval canal cross-sections than the apical third. The latter point needs to be stressed, as the weakness of traditional file systems is the under-preparation of the coronal and middle thirds of canals that anatomically demonstrate more oval cross-sections and greater tapers in the buccolingual dimension. Furthermore, the increase in preparation size benefits disinfection (30) and, although the XPS groups resulted in more enlargement, no significant differences were observed in the apical third. Hence, it is of little consequence to the overall cleaning efficacy that no significant differences were found between the experimental groups XPS15 and XPS25, and between them and the PTNX3 group, respectively.

Recently, two studies compared XPS and PTN regarding their shaping ability. The first study (31) compared the systems following the manufacturer's instructions of use employing cone-beam computed tomography in preparing straight single-rooted permanent teeth with three different pre-created canal taper sizes: 30, 35, and 40. The study's findings have shown that the XPS removed more dentine and demonstrated more prepared areas than PTN (30/.07) in almost all levels and apical sizes, assuming that the XPS might expand more than 0.04 taper in large root canals. Our findings confirmed these assumptions for oval canals.

The second study (32) compared the shaping ability of XPS plus an additional 45 s of activation time as suggested elsewhere (19) with that of PTN (40/0.6v) following the manufacturer's instructions. After preparing long oval root canals of mandibular incisors using micro-CT imaging technology, the authors concluded that both systems had similar root canal shaping abilities. Lower anteriors are often very wide in a buccolingual dimension relative to the mesiodistal dimension. In principle, the XPS system instruments oval canals in 2 ways: by rotational extension of the serpentine shape in the direction of the long oval extension, as well as by an eccentric wobble of the tip in more expansive canal areas, allowing for a further reach of the more coronal portions of the file. In a lower anterior, where canal space in the mesiodistal dimension is commonly very confined, this eccentric movement may be limited by the restricted dimensions, thus resulting in less overall surface area being prepared and explaining the authors' results.

From the perspective of mechanical preparation in this study, XPS25 resulted in a significantly smaller percentage of unprepared areas than XPS15 overall and the coronal third. Also, XPS25 removed significantly more dentine than XPS15 overall and in all three thirds. Based on the fact that a positive relationship exists between remaining tooth structure and fracture resistance (29), careful deliberation should be applied whether to resort to fewer preparation movements and shorter preparation times, which might be clinically sufficient and more conservative in dentine removal, or to increase the number of movements and preparation time to improve mechanical cleaning in oval-shaped canals, such as in distal roots of mandibular molars. Further studies are recommended to assess the effects of prolonged XPS use beyond 15 movements after WL was reached regarding residual fracture resistance and the reduction of the microbial load.

## Conclusion

The findings of the study demonstrated that a greater number of movements to the WL increased the ability of XPS to mechanically prepare greater root canal areas. The addition of 10 extra movements significantly reduced the percentage of unprepared areas and increased dentine removal.

### Disclosures

**Acknowledgement:** We thank the Penn Center for Musculoskeletal Diseases (PCMD) NIH/NIAMS P30-AR069619 for the opportunity to utilize their micro-CT imaging systems.

**Conflict of interest:** The authors deny any conflict of interest.

**Ethics Committee Approval:** This study was approved by a panel from Pedro Ernesto University Hospital/Rio de Janeiro State University Ethical Committee (CAAE n° 62903316.3.0000.5259/CEP-HUPE).

**Peer-review:** Externally peer-reviewed.

**Financial Disclosure:** The work was supported by the Coordenação de Aperfeiçoamento de Pessoal de Nível Superior, Brazil (CAPES), Finance Code 001 (PDSE-88881.131856/2016-01), and by The University of Pennsylvania Department of Endodontics Research fund.

**Authorship contributions:** Concept – A.P., F.C.S., B.K.; Design – A.P., F.M., F.C.S.; Supervision – F.C.S., B.K.; Funding - B.K.; Materials - B.K.; Data collection &/or processing – A.P., W.J.L.T.; Analysis and/or interpretation – A.P., W.J.L.T.; Literature search – A.P., F.M.; Writing – A.P., F.M., F.C.S.; Critical Review – A.P., W.J.L.T., F.M., F.C.S., B.K.
